# A Perturbed Postural Balance Test Using an Instrumented Treadmill – Precision and Accuracy of Belt Movement and Test-Retest Reliability of Balance Measures

**DOI:** 10.3389/fspor.2021.688993

**Published:** 2021-08-27

**Authors:** Kim J. Lesch, Jere Lavikainen, Vesa Hyrylä, Paavo Vartiainen, Mika Venojärvi, Pasi A. Karjalainen, Heikki Tikkanen, Lauri Stenroth

**Affiliations:** ^1^Institute of Biomedicine, Sport and Exercise Medicine, University of Eastern Finland, Kuopio, Finland; ^2^Department of Applied Physics, University of Eastern Finland, Kuopio, Finland

**Keywords:** postural balance, instrumented treadmill, perturbation, reliability, accuracy, precision

## Abstract

A perturbed postural balance test can be used to investigate balance control under mechanical disturbances. The test is typically performed using purpose-built movable force plates. As instrumented treadmills become increasingly common in biomechanics laboratories and in clinical settings, these devices could be potentially used to assess and train balance control. The purpose of the study was to investigate how an instrumented treadmill applies to perturbed postural balance test. This was investigated by assessing the precision and reliability of the treadmill belt movement and the test-retest reliability of perturbed postural balance test over 5 days. Postural balance variables were calculated from the center of pressure trajectory and included peak displacement, time to peak displacement, and recovery displacement. Additionally, the study investigated short-term learning effects over the 5 days. Eight healthy participants (aged 24–43 years) were assessed for 5 consecutive days with four different perturbation protocols. Center of pressure (COP) data were collected using the force plates of the treadmill while participant and belt movements were measured with an optical motion capture system. The results show that the treadmill can reliably deliver the intended perturbations with <1% deviation in total displacement and with minimal variability between days and participants (typical errors 0.06–2.71%). However, the treadmill was not able to reach the programmed 4 m/s^2^ acceleration, reaching only about 75% of it. Test–retest reliability of the selected postural balance variables ranged from poor to good (ICC 0.156–0.752) with typical errors between 4.3 and 28.2%. Learning effects were detected based on linear or quadratic trends (*p* < 0.05) in peak displacement of the slow forward and fast backward protocols and in time to peak displacement in slow and fast backward protocols. The participants altered the initial location of the COP relative to the foot depending on the direction of the perturbation. In conclusion, the precision and accuracy of belt movement were found to be excellent. Test-retest reliability of the balance test utilizing an instrumented treadmill ranged from poor to good which is, in line with previous investigations using purpose-built devices for perturbed postural balance assessment.

## Introduction

Human postural balance has been defined as the ability to sustain an upright posture (Papengaaij et al., 2014). Low et al. (2017) defined postural control as maintaining, achieving, or restoring postural balance despite executable tasks. Sufficient postural control is crucial for executing activities of daily living. Thus, postural control has an important role in everyday life (Jancova, 2008; Anson et al., 2017). Postural control requires the integration and smooth coordination of multiple sensorimotor systems, namely, visual, vestibular, somatosensory, and higher-level premotor, and motor systems (Mancini and Horak, 2010). Impaired postural control may result in falls because of loss of balance. Around one-third of people aged over 60 years fall yearly, and fall risks increase substantially with advancing age (Gerards et al., 2017). Neurological and musculoskeletal disorders deteriorate postural control, thus having a negative effect on safe mobility (Mancini and Horak, 2010). Therefore, maintaining and improving postural control and balance are an essential goal of clinical interventions (de Jong et al., 2020), and research is needed to support the development of effective interventions.

Based on a traditional definition, balance control can be divided into static balance control in which the center of mass movements maintains the balance, while the base of support remains stationary; and dynamic balance control in which both the center of mass and base of support are moving (Shumway-Cook and Woollacott, 2016). This traditional definition does not capture all the important aspects of balance control; thus, Shumway-Cook and Woollacott (2016) suggest postural balance control to be divided into four types: static steady-state balance: maintaining a steady position while sitting or standing; dynamic steady-state balance: maintaining a steady position during movements such as walking; proactive balance: anticipation of a predicted postural disturbance; reactive balance: response to an unpredicted postural disturbance. Numerous postural balance tests exist in clinical use such as the Berg Balance Scale (BBS) and Timed Up and Go (TUG). These tests are easy and quick to perform and thus, are often used in clinical practice. However, they may be subjective, lack responsiveness to small changes, and are not always sensitive enough to detect early deterioration in postural balance or changes due to interventions (de Jong et al., 2020). Moreover, these tests simultaneously assess many of the above-mentioned four types of balance control but provide little information for research on mechanisms of balance improvements or targets for practical interventions.

Recently, mainly because of technological development, computerized dynamic posturography with purpose-built devices has been increasingly used for measuring postural balance. These devices typically consist of a force plate on top of a movable platform which allows perturbation applied through the base of support. These computerized posturography devices measure the adaptive mechanisms of the whole postural control system including sensory, motor, and central mechanisms (Yuntao et al., 2017). The benefit of these devices is that they can assess multiple aspects of balance, namely, static steady-state, proactive, and reactive balance. A drawback of typical computerized dynamics posturography is that the test is performed in a standing posture, but most falls occur during walking or sit-to-stance transfers. Still, balance control under perturbed standing conditions predicts future falls (Sturnieks et al., 2013), and training on perturbed standing conditions reduces fall incidences (Rosenblatt et al., 2013). Thus, the controlled environment that a standing condition provides has a value in both balance testing and training contexts despite not being the particular task in which falls typically occur.

Purpose-built computerized dynamic posturography devices can only be used for a single purpose, which makes them costly investments for research institutes but, unlike clinical tests, allow the measurement of a specific aspect of postural balance performance,. There has been an increase in the use of treadmills with integrated force sensors (i.e., instrumented treadmills) for the investigation of human locomotion, thus, this type of treadmill has become accessible for an increasing number of researchers and clinical practitioners. Instrumented treadmills can measure the required parameter for postural balance assessment, namely, the center of pressure (COP). Additionally, they can be used to perturb balance. Therefore, they provide instrumentation for performing dynamic posturography measurements to assess static and dynamic steady-state, proactive, and reactive balance with devices already existing in many laboratories. However, the reliability and validity of instrumented treadmills for postural balance measurements have been questioned. Instrumented treadmills are susceptible to errors especially in ground reaction force and COP measurements (Sloot et al., 2015) because of mechanical noise or vibrations induced by the treadmill structure to the sensors (Willems and Gosseye, 2013). On the other hand, Fortune et al. (2017) showed that the COP measurement accuracy of an instrumented treadmill can be on par with a traditional ground-mounted force plate, and Collins et al. (2009) showed that COP error can be reduced to a similar level compared with a ground-mounted force plate using a calibration procedure. There can be also differences in the accuracy between devices from different manufacturers to deliver perturbations, which Crenshaw et al. (2019) speculated to be due to unique control strategies and computations. Nevertheless, encouraging results were provided by a preliminary feasibility study conducted by Yuntao et al. (2017), who evaluated the use of an instrumented treadmill (FTM-1200WA; Tec Gihan, Kyoto, Japan) as a standing postural balance measurement device. The study indicated that the reliability of the treadmill-based measurement is comparable with that of computerized dynamic posturography measurement using a purpose-built device (MPS-3102; Balance Master, NeuroCom, Clackamas, USA; ICC *r* = 0.67–0.7). In contrast, results obtained using the instrumented treadmill and purpose-built device differed substantially.

The purpose of this study was to examine if an instrumented treadmill in combination with an optical motion capture system can be used to assess perturbed postural balance. This study concentrated on reactive postural balance with a proactive component included in the assessment as the direction of perturbation was known and the perturbation could be anticipated although exact timing was unknown. COP trajectory in the antero-posterior direction was used as the outcome measure. Following a previous study utilizing a purpose-built perturbed postural balance assessment device (Piirainen et al., 2013), we tested the balance with four perturbation protocols (slow and fast, forward and backward directions). From a theoretical point of view, it is of interest to include perturbations in both directions as the balance maintenance requires the use of different muscle groups when recovering from the perturbation in different directions and it may involve different balance strategies such as ankle strategy and hip strategy.

To this end, we performed a between-days test–retest study that allowed us to evaluate the reliability of the balance assessment as well as short-term learning effects over 5 days. We defined changes that occurred between days as short-term learning, whereas acute changes that occurred within a day were considered as habituation. We hypothesized that: (1) the instrumented treadmill can be used to induce perturbations of the base of support precisely, accurately and reliably, (2) the parameters calculated from the COP trajectory to quantify balance performance show similar reliability as previously reported for purpose-built devices, and (3) learning is observed in balance performance over 5 days. If the study supports the hypotheses, instrumented treadmills in combination with an optical motion capture system could provide a tool to analyze perturbed postural balance in research settings. Furthermore, this setup can be potentially used for postural balance training with continuous monitoring of the progression of balance performance.

## Methods

### Participants and Protocol

Eight people without current musculoskeletal pain or physical limitations volunteered for the study (two females, six males, aged between 24 and 43 years, body mass 64.2–105.6 kg). They were informed about the study, testing protocols, and the use of data according to the institutional guidelines.

Testing was conducted for 5 consecutive days with an identical test setup each day. Four test protocols were commenced with a single protocol and included 10 perturbations with a given direction (backward or forward) and speed (slow or fast) at random intervals. Each day, the protocols were performed in the same order: slow backward, fast backward, slow forward, and fast forward. Participants were made aware of the perturbation direction and speed before commencing the test. Each protocol was performed twice. The first performance was considered as habituation, and the results were calculated from the second performance. Habituation was performed to accustom the participants to the protocol and to mitigate the potential order effect. Stepping response was not allowed, and the habituation trial successfully removed the need for taking a step to maintain balance, which was occasionally observed in the habituation trial but there was none in subsequent trials. Habituation was included in the test setup each day to keep the test setup the same for the examination of the reliability between days. The perturbation intervals were different for habituation and the measurement protocol, but the same across participants and days. The slow protocols lasted, in total, 48–49 s depending on the direction, and the fast protocols lasted 52–53 s. The delay between perturbations was 4.5 ± 0.9 s (mean ± SD). The delay was confirmed to be sufficient for recovering a stable balance between the perturbations. During the measurements, we confirmed that COP recovered close to the initial location and that the COP trajectory was stable before a new perturbation was delivered.

**Figure 1:**
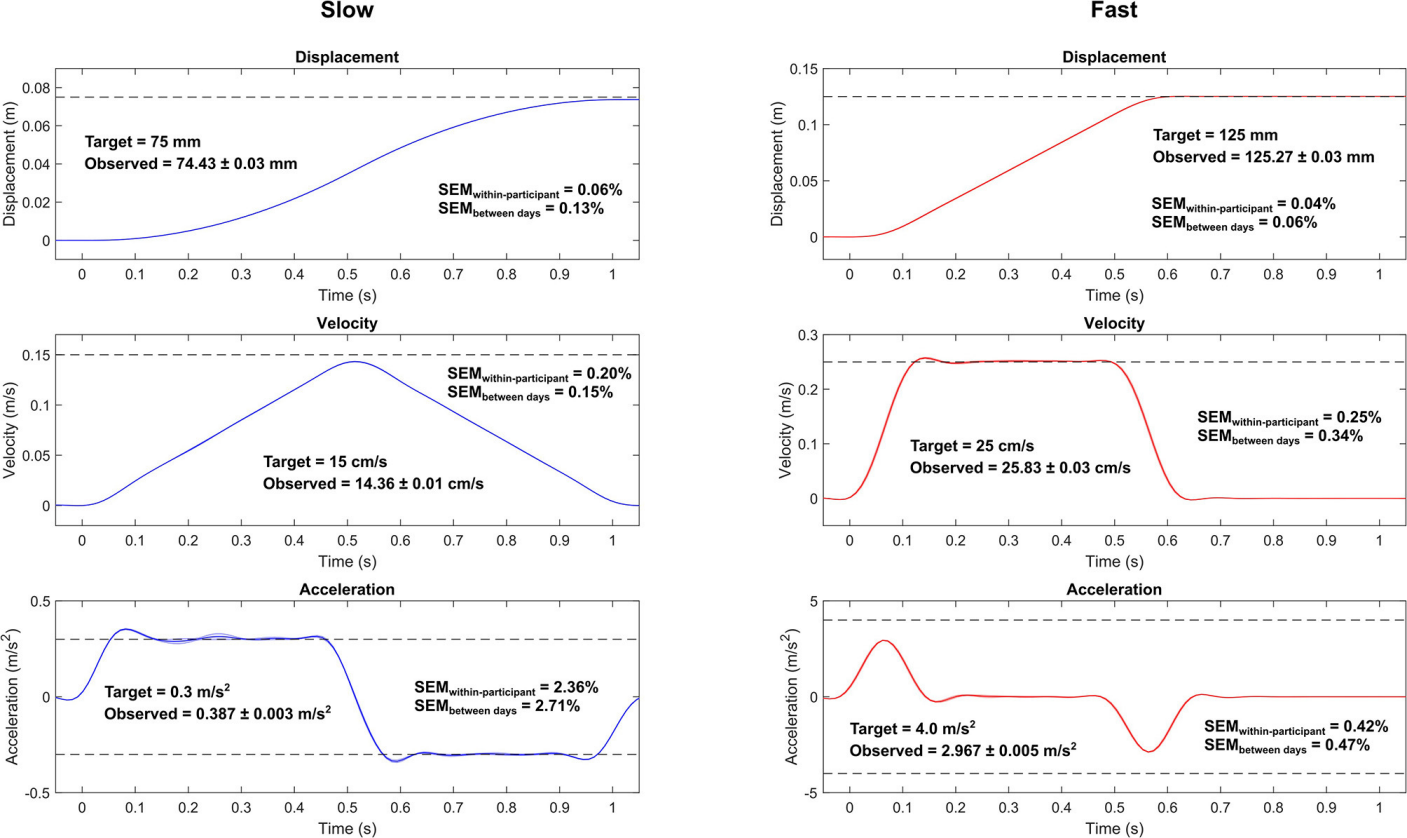
Measured belt movement and its reliability metrics for the slow (left) and fast (right) protocols. The curves represent the mean and between-participant standard deviation (shaded area; note that the standard deviations are extremely small). Only the backward perturbations are shown, since the patterns are identical in both directions. Dashed vertical lines show the target values of the peak displacement, velocity, and acceleration based on programmed control signals of the belt. Time zero indicates the identified start of the perturbation. Note that the time scales are the same for both protocols, but the vertical scales differ. The observed values are presented as the mean and within-participant standard deviation of the peak values. SEM_within−participant_, within-participant standard error of measurement; SEM_between days_, between-day standard error of measurement.

Initially, the participants stood barefoot on a split-belt instrumented treadmill (M-gait, Motek Medical, Houten, The Netherlands) with feet pointing forward with a standardized width of 30 cm (center to center distance) both feet on different force plates/belts, hands on the sides of the body, and gaze fixed to a point at the level of the eyes on the opposing wall. A previously published (base of support) movement pattern (Piirainen et al., 2013) was implemented using the D-Flow software (Motek Medical, Houten, The Netherlands) controlling the treadmill. The test setup comprised four protocols. A single protocol included only slow or fast perturbations in one direction. The software allows setting the target velocity for the belt, the maximal acceleration that the motor can utilize, and the duration that the belt is driven with the target velocity. In slow perturbations, the belt was set to move with a maximal acceleration (and deceleration) of 0.3 m/s^2^ targeting 0.15 m/s belt velocity for 0.5 s resulting in a ramp-like velocity profile without plateau (Figure 1). The resulting calculated ideal belt movement was 75 mm. For fast perturbations, the target speed was set to 0.25 m/s for 0.5 s, while the maximal acceleration was limited to 4 m/s^2^ and then decelerated to a full stop with the same acceleration. The resulting calculated ideal belt movement was 125 mm. Unlike in the study of Piirainen et al. (2013) in which electromechanical cylinders could move the force plate forward and backward, the opposite directions of perturbations in this study were enabled by changing the direction the subjects were facing, i.e., the belt only moved in one direction. This is a limitation of the system that can be overcome by updating the software and may not apply to all corresponding systems.

### Data Analysis

To account for the relative movement of the belt (base of support) and the treadmill structure (force plate), the movement of the belt was recorded by tracking three reflective markers placed on the treadmill using an optical motion capture system (100 Hz, Vicon Vero, Vicon Motion Systems Ltd., Oxford, United Kingdom), while the COP was measured with the instrumented treadmill (1,000 Hz). Measurement of the belt movement allowed us to express the COP trajectory relative to the base of support similarly as in the case where the force plate would be moving. The optical motion capture system was also used to measure the location of four markers on each foot (big toe, heel, and medial and lateral malleolus). The malleolus markers were used to express the COP location relative to the ankle joint center. This information can be used to evaluate potential anticipation of the coming perturbations by shifting the COP location toward the toes in case of forward perturbation or toward the heel in case of backward perturbation. Heel and toe markers can be used to express the COP trajectory relative to foot length, but here we chose to report the results in absolute units consistent with a previous study (Piirainen et al., 2013).

**Figure 2:**
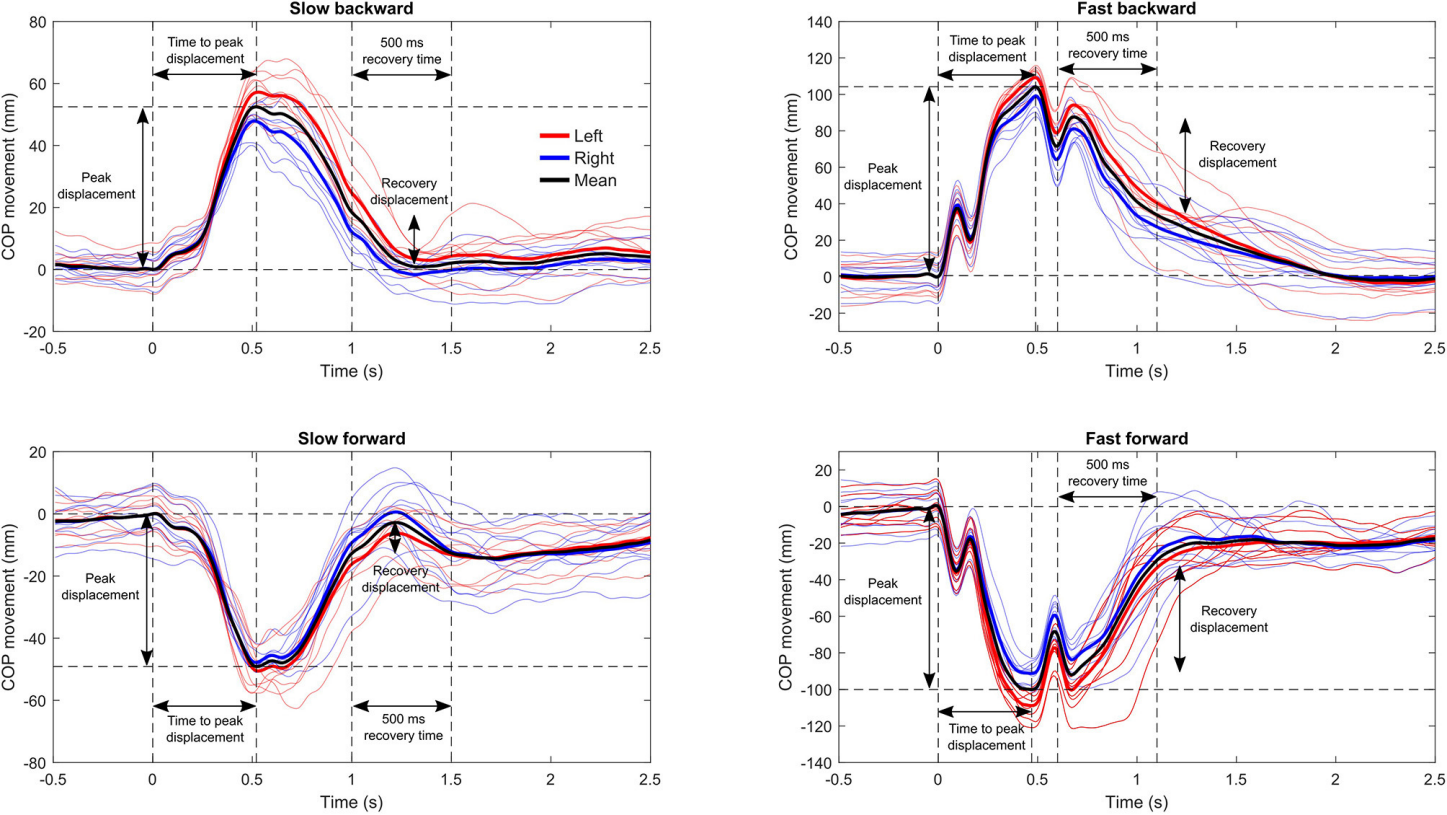
Example of the center of pressure (COP) trajectories relative to the belt during the four perturbation protocols from a single participant on a single session. The thin lines represent the response to each perturbation, and the thick lines are the ensemble average trajectory for each leg. The outcome measures were calculated from the ensemble average trajectory of both legs (black) and alternatively from each response to perturbation separately (average of right and left legs) with subsequent averaging of the results to obtain the final outcome. The horizontal dashed lines denote the peak displacement and the zero displacement level, and the vertical lines mark the instance of perturbation onset, time at peak displacement, and 500-ms post peak displacement for the calculation of recovery displacement.

COP and marker data were filtered using a fourth-order zero-lag 5 Hz low-pass Butterworth filter, and COP data were interpolated to 100 Hz to match the sampling frequency of motion capture data. In the analysis, we only considered the anteroposterior direction of the COP trajectory. The displacement of the COP relative to the base of support was calculated by subtracting the COP displacement from the belt displacement. The onset and the end of the perturbations were detected from the marker-based belt velocity profile using 3 and 2 cm/s thresholds for onset and end detections, respectively, followed by constant time shifts for locating the actual onset and end that depended on the protocol (slow or fast). Three outcome measures reported by Piirainen et al. (2013) were quantified from the COP trajectories: peak displacement, time to peak displacement, and recovery displacement, which allowed the results to be compared with those measured using a purpose-built movable force plate system. Peak displacement and time to peak displacement were defined as the peak of the COP trajectory relative to position at the instance of perturbation onset, and the time to the peak, respectively. Recovery displacement was defined as the peak-to-peak displacement of the COP trajectory during a 500-ms time window after the end of belt movement. The 500-ms recovery period has been used previously by Piirainen et al. (2013) and Chien and Hsu (2018), with the authors of the latter study justifying the selection by averaged active response time observed in previous studies. Additionally, we calculated the COP location relative to the ankle joint center (the midpoint between medial and lateral malleolus) at the instance of perturbation onset (initial COP location) to evaluate potential anticipation behavior. The extraction of the outcomes from the COP data was done with two different approaches: from ensemble average trajectory and individual trajectories. For the extraction of the outcomes from the ensemble average COP trajectory, the COP trajectory was cut into sections defined by the above-mentioned onset and end detention, and ensemble average COP trajectory was calculated for the left and right legs to improve the signal-to-noise ratio of the data. The trajectories were set to zero at the instance of perturbation onset and, finally, the mean trajectory of the left and right leg COP trajectories was calculated. Then, the three outcome measures were determined from this average trajectory (Figure 2). Additionally, extraction of the outcome measures was performed from each response to the perturbations separately (average of right and left legs), and the final outcome was calculated as the average of the outcomes from individual trajectories. Body mass was calculated by dividing the mean vertical force recorded during the trial by 9.81 m/s.

### Statistical Analysis

The reliability of the postural balance outcomes calculated from the COP trajectory was evaluated using intraclass correlation (ICC), a measure of relative reliability and standard error of measurement (SEM), a measure of absolute reliability (Weir, 2005). For ICC calculation, we used a single rater two-way random-effect model for absolute agreement (ICC 2.1). ICC values were interpreted according to Koo and Li (2016) with the following cutoff points: < 0.5 poor, 0.5–0.75 moderate, 0.75–0.9 good, and >0.9 excellent reliability. SEM was calculated by repeated measured analysis of variance (ANOVA) that partitions the observed variability to the variability arising from between-days and within-day effects. The within-day variability is further partitioned into between participants and error variability. The error variability is an estimate of the variability within-day that is not accounted for by between-participant differences and, therefore, estimates the random variability within-participant. By taking the square root of the mean square within-day error, we estimated the typical measurement error (Weir, 2005) and later referred to this as SEM_within−participant_. For reliability analysis of the belt movement, in addition to the SEM_within−participant_, we reported the estimate of typical between days difference (standard deviation between days), which was calculated by taking the square root of the between days mean squares. We referred to this later as SEM_betweendays_. The SEM values are presented as the percentage of the mean and, additionally, in original units in the supplement material for the reliability of postural balance outcomes. To investigate if the body mass of the participant affected the movement of the belt, we calculated Pearson correlation coefficients of belt peak displacement, velocity, and acceleration with body mass. In this analysis, we utilized the data from all days and both perturbation directions within a speed resulting in 80 observations (forward and backward protocols, 5 days, and eight participants) for each analysis. Pooling the data was justified by the assumption that the influence of body mass is much larger than any potential effect of measurement day or standing direction of the participant; thus, each trial could be considered as an independent observation. In case of a significant correlation, linear regression analysis was performed to determine the magnitude of the effect that the body mass had on the belt movement. Learning effects were assessed using repeated-measures ANOVA comparing the results obtained on different days followed by an additional analysis of linear and quadratic trends to assess systematic patterns in the values observed on different days. A linear trend was considered to model a situation in which learning is occurring throughout the 5 days, whereas a quadratic trend was considered to model learning with a ceiling effect during the 5 days. Reliability and learning effect analyses were performed in the IBM SPSS Statistics software (version 27, SPSS Inc., IBM, Armonk, NY, United States), and the correlation and regression analysis between belt peak displacement, velocity, and acceleration and participant body mass (derived from the force data) was performed in MATLAB (R2019b, The MathWorks, Inc., Natick, MA, United States). The statistical significance was set at *p* < 0.05.

## Results

During the data analysis, we noticed that the first perturbation in a set was systematically different from the rest of the perturbations in the set, showing higher acceleration, especially in the slow protocol (Supplementary Figure 1). Hence, we removed the first perturbation from the analyses and calculated all outcome measures based on the remaining nine perturbations in the set. The movement of the belt was highly repeatable and accurately followed the control signal. In both the slow and fast protocols, the displacement of the belt was always less than half a millimeter from the target value (Figure 1). Belt peak velocities showed < 1 cm/s error. The largest deviation from the target values was observed in peak acceleration in the fast protocol in which the belt reached about 75% of the target value. In the slow protocol, peak accelerations overshot the target by an average of 29%. The largest within-participant and between-day standard errors in the belt movement were observed in the peak accelerations of the slow protocol in which these errors were <3% of the mean. All the other SEMs were <1% of the mean.

We did not observe statistically significant correlations between participant body mass and the measured peak displacements or velocities, but a weak correlation was observed between body mass and peak acceleration (slow protocol *r* = 0.262, *p* = 0.019; fast protocol *r* = 0.298, *p* = 0.007, Supplementary Figure 2). Regression analysis indicated that with each 1 kg increase in body mass, the peak acceleration would increase by 0.1% in both the slow and fast protocols.

**Figure 3:**
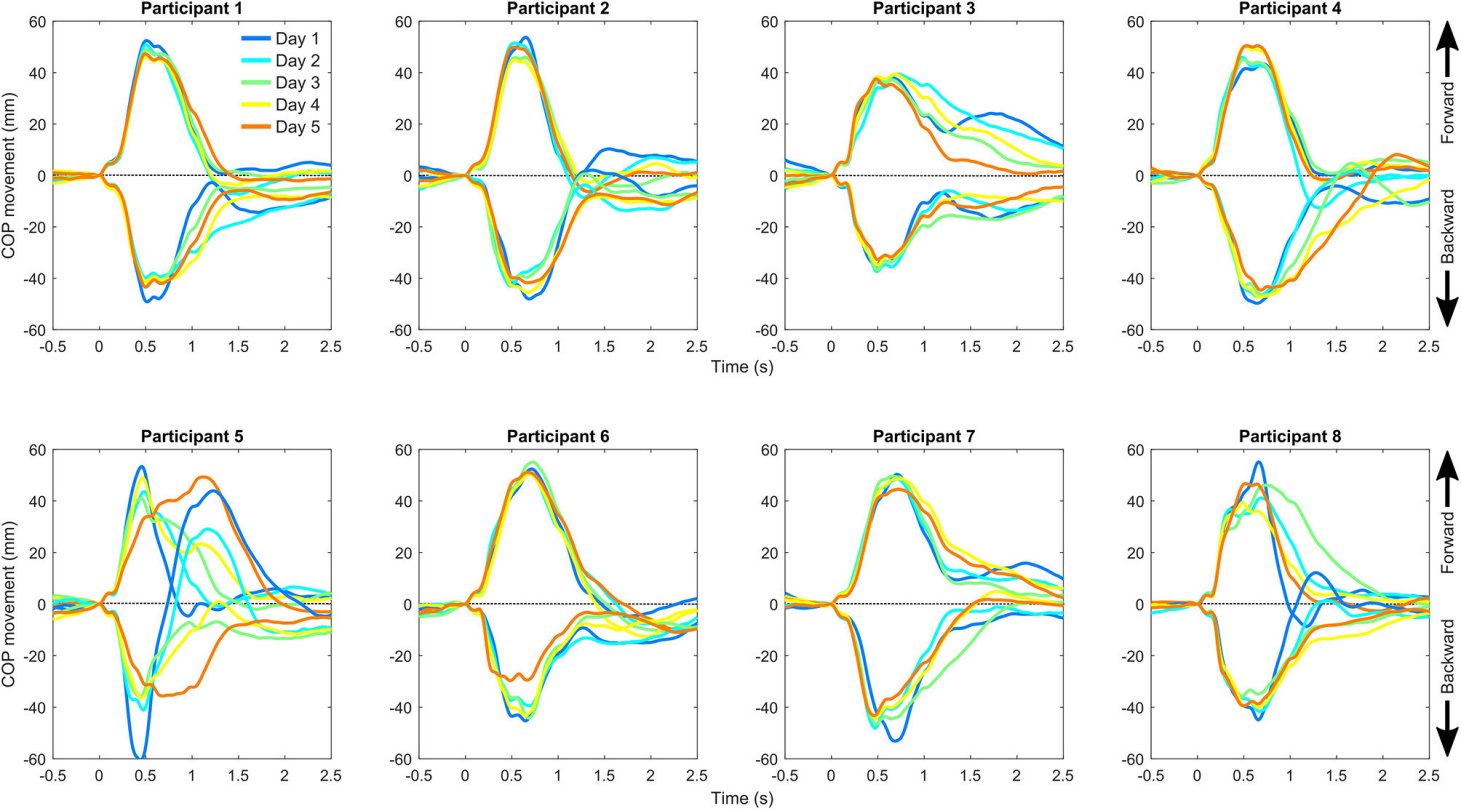
Center of pressure (COP) movement relative to the belt in the slow backward and forward perturbation protocols separately for each participant and measurement day. The positive direction of the center of pressure movement is forward (direction of gaze) and occurs in response to backward perturbation of the belt.

**Figure 4:**
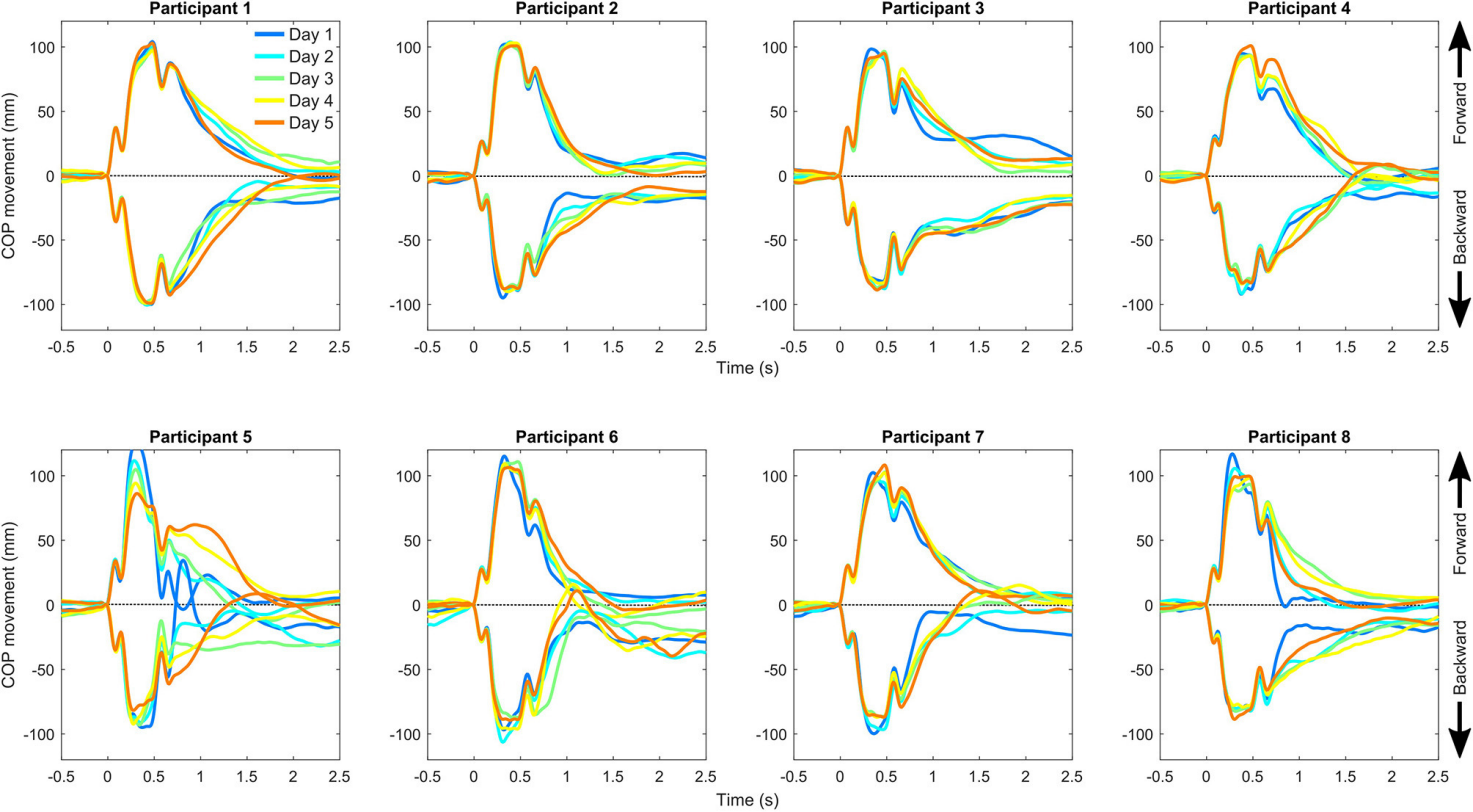
Center of pressure (COP) movement relative to the belt in the fast backward and forward perturbation protocols separately for each participant and measurement day. The positive direction of the center of pressure movement is forward (direction of gaze) and occurs in response to backward perturbation of the belt.

Visual observations indicated that COP trajectories show repeatable patterns between days (Figures 3, 4). One participant (participant 5) showed clearly different COP movement patterns in both slow protocols for day 5 compared with other days. This probably reflects an altered balance maintenance strategy for day 5. We excluded the participant from the reliability analyses of the slow protocols, as these vastly different results would have inflated the reliability metrics (Table 1). This result probably reflects an altered balance maintenance strategy. The reliability results using the whole dataset are provided in Supplementary Table 1. Overall, the reliability results were not markedly influenced by the analysis methods, i.e. if the outcomes were calculated from the ensemble average COP trajectory or individual trajectories and subsequently averaged. The absolute reliability (SEM) of time to peak displacement and recovery displacement was better when the perturbation direction was backward compared to forward. Based on ICC values the reliability in different variables and perturbation directions and speeds ranged from poor to good.

**Table 1:** Test–retest reliability of the selected outcome measures describing perturbed postural balance performance.

		**Slow backward**	**Slow forward**	**Fast backward**	**Fast forward**
**Based on ensemble average trajectory**					
Peak displacement (mm)	ICC	0.598 (0.277–0.893)	0.571 (0.238–0.882)	0.252 (0.015–0.671)	0.547 (0.247–0.854)
	SEM	3.30	2.69	6.27	4.13
	SEM%	7.00	6.32	6.16	4.56
Time to peak displacement (s)	ICC	0.503 (0.187–0.854)	0.506 (0.183–0.857)	0.549 (0.228–0.856)	0.549 (0.228–0.856)
	SEM	0.06	0.07	0.04	0.05
	SEM%	9.93	11.78	8.90	12.66
Recovery displacement (mm)	ICC	0.719 (0.430–0.932)	0.669 (0.346–0.918)	0.738 (0.468–0.929)	0.445 (0.153–0.803)
	SEM	3.70	4.64	7.65	13.31
	SEM%	18.14	27.65	15.83	28.22
**Based on individual trajectories**					
Peak displacement (mm)	ICC	0.638 (0.326–0.907)	0.574 (0.235–0.884)	0.349 (0.083–0.743)	0.513 (0.215–0.838)
	SEM	2.73	2.41	5.53	3.97
	SEM%	5.56	5.41	5.32	4.28
Time to peak displacement (s)	ICC	0.388 (0.069–0.804)	0.156 (−0.098 to 0.653)	0.562 (0.255–0.861)	0.550 (0.237–0.858)
	SEM	0.06	0.08	0.03	0.04
	SEM%	9.82	12.65	7.55	8.37
Recovery displacement (mm)	ICC	0.752 (0.478–0.942)	0.658 (0.336–0.915)	0.680 (0.389–0.909)	0.438 (0.147–0.800)
	SEM	3.29	4.47	7.26	13.23
	SEM%	14.96	23.65	14.34	25.91

*Outcome variables are calculated from ensemble average COP trajectory and alternatively from each individual trajectories*.

*ICC, intraclass correlation and 95% confidence interval; SEM, standard error of measurement expressed as percentage of the mean. SEM reported here refers to the SEM_within−participant_ explained in the statistical analysis chapter. Data from one participant on one day was excluded from the slow protocols*.

**Figure 5:**
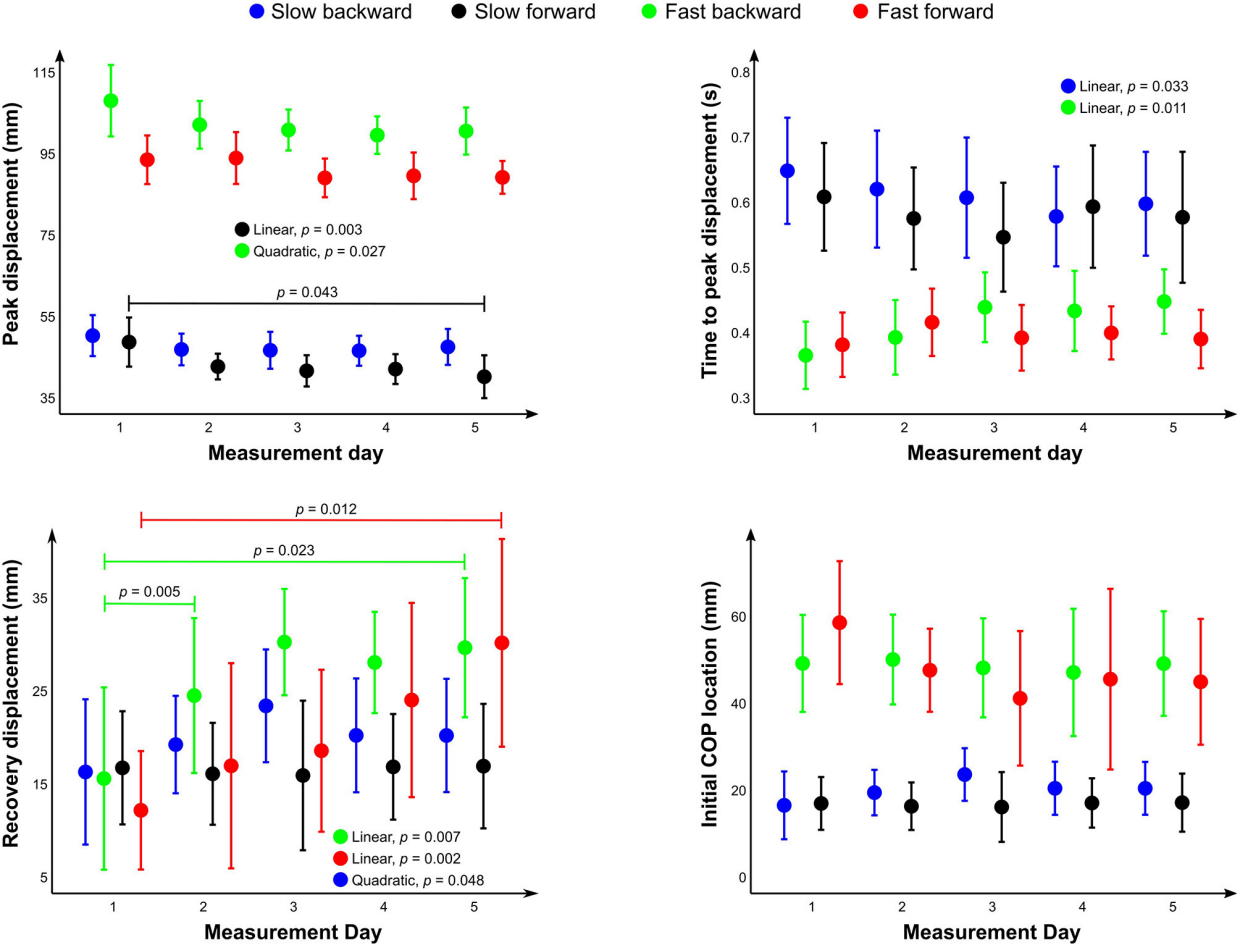
Mean values of the different outcome measures and center of pressure location at the instance of perturbation onset relative to the ankle joint. Whiskers represent the 95% confidence interval of the mean. Statistically significant differences between days and statistically significant linear or quadratic trends across the days are shown.

Over the five consecutive testing days, time to peak displacement showed a linearly increasing trend in the slow backward (*p* = 0.033) and a linearly decreasing trend in the fast backward (*p* = 0.011) protocols (Figure 5). Peak displacement showed a linearly decreasing trend in the slow forward protocol (*p* = 0.003) and a quadratic trend in the fast backward protocol (*p* = 0.027) with an initial decrease as a function of time. Additionally, peak displacement from day 1 significantly differed from day 5 in the slow forward protocol (*p* = 0.043). The COP was located approximately 4–5 cm anterior from the ankle joint center. The COP location was systematically approximately 1 cm more from the anterior in the forward perturbation protocols, which is related to the fact that the participants were aware of the perturbation direction and anticipated it by moving the COP location anteriorly in case of forward or posterior perturbation in case of backward perturbation to provide a possibility for a larger movement amplitude of the COP. COP location relative to the ankle joint at the instance of perturbation onset showed a quadratically decreasing trend (i.e., COP was closer to the ankle joint on later days) in the slow forward protocol (*p* = 0.021). Most of the decrease occurred between days 1 and 2.

## Discussion

The purpose of this study was to examine the capability of an instrumented treadmill for testing perturbed standing postural balance. We hypothesized that the instrumented treadmill is precise and accurate in delivering intended perturbations and that the measured outcomes show comparable values with previously reported ones using purpose-built devices and with comparable test–retest reliability. Additionally, we examined potential short-term learning effects that are important to acknowledge when designing longitudinal studies and provided indications if the system could also be useful as a balanced training method. The results indicate that the treadmill can repeatedly deliver perturbations with low between-session and between-participant variations in displacement, speed, and acceleration. Postural balance evaluated with the treadmill in combination with a motion capture system (Figure 5) showed comparable results with Piirainen et al. (2013) using a purpose-built device (numeric values not given, data provided as a bar chart). The only marked difference between the studies was the recovery displacement of the fast perturbation protocol in which the results of this study were about half of those observed in the study of Piirainen et al. (2013). The observed test–retest reliability was also on par with the previous report using purpose-built devices (Yuntao et al., 2017). Finally, the analysis provided evidence for short-term learning effects on multiple outcome measures. In some variables, the results seemed to plateau within 5 days; whereas in others, continued learning effects were observed throughout 5 days. Overall, the study supports the usability of an instrumented treadmill in combination with a motion capture system for testing perturbed postural balance.

### Accuracy and Reliability of Treadmill Belt Movement

One of the aims of the study was to quantify the precision, accuracy and reliability of the belt movement for delivering perturbations. The belt movement was highly repeatable and accurately replicated the target velocity. In the fast protocol, the belt reached only about 75% of the maximal allowed acceleration. The reason for this was the limit in the rate of rising of the acceleration. However, regardless of not reaching the maximal allowed acceleration set for this protocol, the peak accelerations were still highly repeatable both within (between days) and between-participants with a typical error of < 0.5% of mean in the fast protocol. Thus, the deviation from the intended acceleration does not invalidate comparisons between sessions and participants. In both slow and fast perturbations, the mean belt displacement during perturbation was within < 1 mm of the calculated target. This is noteworthy since the controls for the belt movement only included target velocity maximal acceleration/deceleration to be used.

We also examined the impact of body mass on treadmill belt movement using correlation analysis and found that body mass did not significantly correlate with the displacement amplitude or peak velocity of the belt, but a significant correlation was observed between body mass and peak acceleration. The observed correlation may be related to the control system of the treadmill and the increased demand for the adjustments of motor torque due to added body mass. Regression analysis showed that a 1 kg increment in body mass had a 0.1% effect on belt peak acceleration. Thus, for example, a 50 kg between-subject variation on body mass is expected to have a 5% effect on belt peak acceleration. The effect is not negligible but is comparable with the within-participant typical error in peak acceleration in the slow protocol. Thus, we consider that the effect that body mass has on belt movement does not invalidate between-participant comparisons. The results regarding belt movement accuracy (< 1% deviation in total displacement from target value) are generally in line with those of a previous report (2–5%) (Crenshaw et al., 2019), although we reported a slightly better accuracy except for peak acceleration in both slow and fast protocols (between 26 and 29% in this study versus ≤ 5% in that of Crenshaw). In addition, Crenshaw et al. (2019) found an effect of body mass on belt displacement and velocity accuracy but not on acceleration. This difference may be due to the use of different treadmills.

Interestingly, we noticed that the belt movement in the first perturbation systematically differed from the rest of the perturbations in the set (Supplementary Figure 1). The reason for this behavior was that a brake is released simultaneously with the start of the first movement of the belt resulting in a slower start of the perturbation followed by abrupt acceleration. This can be avoided by adding a period of zero velocity at the beginning of the control script that releases the brake.

### Reliability of the Perturbed Postural Balance Outcome Measures

Test–retest reliability of the selected outcome measures of balance performance was mostly moderate and not markedly affected by the calculation of the outcomes from the ensemble average COP trajectory or individual trajectories (Table 1). In time to peak displacement, the relative reliability (ICC) was better when calculated from the ensemble average COP trajectory compared with the calculation from individual trajectories, but the calculation type did not affect absolute reliability (SEM). Poor reliability based on ICC was observed in peak displacement of the fast backward protocol and recovery displacement of the fast forward protocol when outcomes were calculated from the ensemble average COP trajectory. Poor reliability was observed in time to peak displacement of the slow backward and forward protocols, peak displacement of the fast backward protocol, and recovery displacement of the fast forward protocol when outcomes were calculated per trajectory. The poor reliability is partly due to observed learning effects, as we used the absolute agreement definition of the ICC calculation as opposed to consistency definition. The low end of the ICC values reported in this study (ICC 0.16) is worse than that which has been reported in previous investigations of perturbed postural balance assessments, which have reported ICCs ranging from 0.61 to 0.96 (Yuntao et al., 2017; Crenshaw et al., 2019). However, it should be noted that the test protocols, devices and outcome measures differ between the studies.

We want to point that visually inspecting the shape of the COP trajectories showed repeatable patterns between repeated perturbation within a session and between days (Figures 3, 4). It seems that there is a COP trajectory “fingerprint” that is somewhat unique to the participant, although the reliability of the selected outcome measures was only modest. The finding also indicates that postural balance correction strategies are relatively stable within a participant. Hence, the modest reliability is probably not related to the instrumentation but has issues with the used outcome metrics. It could be of interest to further investigate individual COP trajectory shapes in future studies and identify outcome metrics that better capture the individual features of COP responses.

### Between-Day Differences and Learning Effect

We quantified potential learning effects by investigating between-day differences and between-day linear and quadratic trends. Statistically significant differences in the postural balance outcome measured were detected only for peak displacement in the slow forward protocol in which the observed peak displacement was larger on day 1 compared with day 5. Statistically significant trends were observed for peak displacement (slow forward and fast backward) and time to peak displacement (slow and fast backward). Both peak displacements and times to peak displacement decreased with time in the slow backward protocol. However, in the fast backward protocol, time to peak displacement increased with time. These are probably a result of short-term learning or habituation. The increase in time to peak displacement in the fast protocol was coupled with a decrease in peak displacement. The result may be due to the participants learning to start the balance-correcting muscle activity earlier, which slows down the anterior COP movement velocity and results in the observed later occurrence of peak displacement.

Interestingly, the perturbation velocity, which was also known by the participants, did not affect the COP location in the backward perturbation condition. In the forward perturbation condition, the COP location was more anterior with the fast perturbation speed. No significant between-day differences were observed in the initial COP location, but there was a significant linearly decreasing trend in the slow forward protocol, which probably indicates habituation to the perturbation protocol allowing the participant to stand with COP closer to the ankle joint center while maintaining balance.

The trends observed in peak displacements and times to peak displacement may indicate short-term learning effects and, therefore, support the use of instrumented treadmills as a potential postural balance training apparatus. However, in this study, perturbation intervals were randomized within the protocol, but the protocol was the same between the days. Hence, it is not clear if the improvements reflect memorizing the protocol or learning in balance control. Earlier studies have shown the importance of task-specific training. Training methods that influence postural balance might be more effective than basic and general exercises (Hrysomallis, 2011; Gerards et al., 2017). Perturbations of the base of support can provide task-specific training and have been named perturbation-based balance training (PBT). The goal of PBT is to improve reactive balance control after destabilizing perturbations (Gerards et al., 2017). PBT performed during walking has been shown to improve perturbed postural balance (Chien and Hsu, 2018). In addition, based on a meta-analysis, PBT seems to be effective for reducing fall risk among older adults and individuals with Parkinson's disease (Mansfield et al., 2015). Future studies could investigate if PBT performed during locomotion is more effective in reducing fall risk than PBT during standing as performed here.

### Comparison With Previous Studies Utilizing Purpose-Built Devices

The perturbation protocols used in this study were based on a previous study by Piirainen et al. (2013). COP peak displacements, times to peak displacement, and recovery displacement showed comparable results with the group of young adults in that study. Moreover, the peak displacements observed in the current study were in line with the study by Walker et al. (2020) which utilized a protocol closely resembling the one used here in the fast condition. The finding suggests treadmill-based perturbed postural balance assessment has good concurrent validity compared with the test performed using a purpose-built device consisting of a commercial force plate driven by electromechanical cylinders. This finding suggests that instrumented treadmills can be utilized for perturbed balance assessments despite the lower accuracy of the COP measured due to mechanical vibrations transmitted to the force sensors and concern regarding the accuracy of movement due to, e.g., belt slackness.

## Limitations

The following limitations related to this study should be acknowledged. First, the small sample size limited the ability of the authors to detect short-term learning effects. With a larger sample size, we could have most probably detected learning effects from more of the parameters. A larger sample size could have also resulted in higher confidence for reliability estimates apparent in reduced confidence intervals. Second, we examined only young and healthy individuals. Thus, reliability estimates for balance outcome measures are not generalizable to other populations, but the technical suitability of an instrumented treadmill for perturbed postural balance measurement is not expected to depend on the population of interest. Third, in this study, the treadmill belt only moved in one direction, which allowed the participants to anticipate perturbation even when the time-lag between perturbations is randomized. However, even when performed with a uni-directional treadmill, the results of the study were in accordance with previous investigations using a multi-directional movable force plate (Piirainen et al., 2013). Also, on average, the difference in COP location relative to the ankle joint center at the instance of perturbation onset was only 1.2 cm. The difference was systematic, so we can conclude that knowing perturbation direction causes anticipation, but the magnitude of anticipation was only around 5% of the total foot length, which is about 10–20% of COP trajectory length in response to the perturbation.

### Suggestions for Future Studies Utilizing Instrumented Treadmills for Perturbed Postural Balance Assessment

The test described in this study can be easily supplemented with measurement of joint kinematics and kinetics (inverse dynamics-based), as the necessary equipment for those measurements are force plates and a motion capture system. Also, adding electromyography measurements, in addition to joint kinematics and kinetics, would allow for a comprehensive assessment of balance maintenance mechanisms. Muscle activity could give more information about the motor control of postural balance by quantification of factors such as anticipatory muscle activity, latency or reaction time, reflective activity, and muscle co-activation. Furthermore, previous studies have coupled measurement with percutaneous electrical stimulation of peripheral nerves to assess H-reflexes during perturbations (Piirainen et al., 2013; Miranda et al., 2019). This measurement can be used to assess spinal sensitivity during postural balance maintenance. When investigating participants with unilateral musculoskeletal conditions or neurological conditions affecting the body asymmetrically, it may be of interest to consider COP trajectories separately for both legs. It may be also of interest to investigate medio-lateral COP movement in response to the perturbations. In future studies, it is advisable to mix directions and speeds of perturbations within a trial when the hardware allows this. This would allow one to include more than 10 perturbations in a trial. In this study, we were able to detect short-term learning effects during the 5 days in some parameters and also observed indications of instantaneous habituation between the training and measurement trials, and within the habituation trial (see details on the Supplementary Material). Future studies should utilize sufficient habituation period to prevent habituation effect biasing the results. At minimum, it needs to be ensured that all experimental groups compared have received equal habituation to the testing procedures. Based on the results of this study, we are not able to give a recommendation on the required amount of habituation, and this aspect should be more thoroughly investigated in the future. We noticed that COP peak displacements occurred around the instance of belt deceleration. This may be because of corrective angular impulse relative to the body center of mass that the base of support deceleration creates. Future studies should investigate protocols in which the velocity plateau is longer and, thus, the base of support deceleration would not help in balance maintenance. Finally, we suggest that COP accuracy during belt movement should be investigated if the information is not available for the particular device. A previous study showed that COP accuracy with an instrumented treadmill depended on belt speed and mass applied on the belt (Fortune et al., 2017).

We noticed that the first perturbation in the trial provides an acceleration profile different from the rest of the perturbations in a trial. The reason for this behavior was that a brake is released simultaneously with the first input to the treadmill. The issue can be resolved in future studies by implementing a short section with zero velocity at the beginning of the protocol.

## Conclusions

The results indicate that an instrumented treadmill combined with an optical motion capture system can be utilized for testing perturbed postural balance similarly as has been previously done using purpose-built motorized force plates. This opens up possibilities for research laboratories and rehabilitation centers with access to such equipment for perturbed balance assessments. However, it should be noted that the results may not be generalized to equipment from different manufacturers. The observed learning effects suggest that the system and protocols can be potentially used for training to improve postural balance, but further research is needed to confirm this. The data presented can be used to inform future studies that will utilize instrumented treadmills for perturbed postural balance assessments regarding required sample sizes and selection of protocols.

## Data Availability Statement

The raw data supporting the conclusions of this article will be made available by the authors, without undue reservation.

## Ethics Statement

Ethical review and approval was not required for the study on human participants in accordance with the local legislation and institutional requirements. Written informed consent for participation was not required for this study in accordance with the national legislation and the institutional requirements.

## Author Contributions

KL, VH, MV, HT, PV, and LS designed the study. KL, JL, PV, and LS conducted the experiments. JL and LS wrote the analysis code. KL, JL, and LS analyzed and interpreted the data. KL and LS wrote the initial manuscript draft. JL, VH, PV, MV, and PK revised the manuscript. HT and PK contributed to funding acquisition and project administration. MV, PV, LS, PK, and HT contributed to supervision. KL, PV, LS, HT, and PK contributed to the development of the infrastructure enabling this study. All the authors read and approved the final manuscript.

## Conflict of Interest

The authors declare that the research was conducted in the absence of any commercial or financial relationships that could be construed as a potential conflict of interest.

## Publisher's Note

All claims expressed in this article are solely those of the authors and do not necessarily represent those of their affiliated organizations, or those of the publisher, the editors and the reviewers. Any product that may be evaluated in this article, or claim that may be made by its manufacturer, is not guaranteed or endorsed by the publisher.
