# Supplementary material for: A Perturbed Postural Balance Test Using an Instrumented Treadmill – Precision and Accuracy of Belt Movement and Test-Retest Reliability of Balance Measures
Source: Front Sports Act Living. 2021 Aug 27;3:688993. doi: 10.3389/fspor.2021.688993 (PMC8429482; doi:10.3389/fspor.2021.688993)
Supplement: Supplementary file 1 [file Data_Sheet_1.PDF]

Supplementary Material

1 Supplementary Tables

Table 1. Test-retest reliability of the selected outcome measures describing the perturbed postural balance performance without removing the clear outlier. Outcome variables were calculated from ensemble average COP trajectory and alternatively from each individual COP trajectory.

		Slow backward	Slow forward	Fast backward	Fast forward
**Ensemble average trajectory**					
Peak displacement (mm)	ICC	0.518 (0.218-0.841)	0.330 (0.077-0.724)	0.252 (0.015-0.671)	0.547 (0.247-0.854)
	SEM	3.43	3.88	6.27	4.13
	SEM%	7.26	9.13	6.16	4.56
Time to peak displacement (s)	ICC	0.089 (-0.110-0.531)	0.454 (0.148-0.812)	0.549 (0.228-0.856)	0.549 (0.228-0.856)
	SEM	0.12	0.08	0.04	0.05
	SEM%	19.93	13.22	8.90	12.66
Recovery displacement (mm)	ICC	0.635 (0.340-0.891)	0.582 (0.266-0.872)	0.386 (0.109-0.764)	0.355 (0.092-0.743)
	SEM	4.11	5.05	6.12	8.55
	SEM%	20.81	30.56	24.08	42.37
**Individual trajectories**					
Peak displacement (mm)	ICC	0.591 (0.293-0.874)	0.335 (0.080-0.727)	0.349 (0.083-0.743)	0.513 (0.215-0.838)
	SEM	2.77	3.56	5.53	3.97
	SEM%	5.61	7.95	5.32	4.28
Time to peak displacement (s)	ICC	0.020 (-0.150-0.449)	0.073 (-0.122-0.517)	0.562 (0.255-0.861)	0.550 (0.237-0.858)
	SEM	0.11	0.08	0.03	0.04
	SEM%	17.31	13.53	7.55	8.37
Recovery displacement (mm)	ICC	0.656 (0.367-0.899)	0.623 (0.310-0.889)	0.403 (0.123-0.775)	0.516 (0.218-0.839)
	SEM	3.73	4.56	5.54	9.50
	SEM%	17.10	23.62	19.74	31.37

ICC, intraclass correlation and 95% confidence interval; SEM, standard error of measurement expressed as a percentage of the mean. SEM reported here refers to the $SEM_{within\text{-}participant}$ explained in the Statistical analysis chapter of the main manuscript. Data from one participant on one day was excluded from the slow protocols.

2 Supplementary Figures

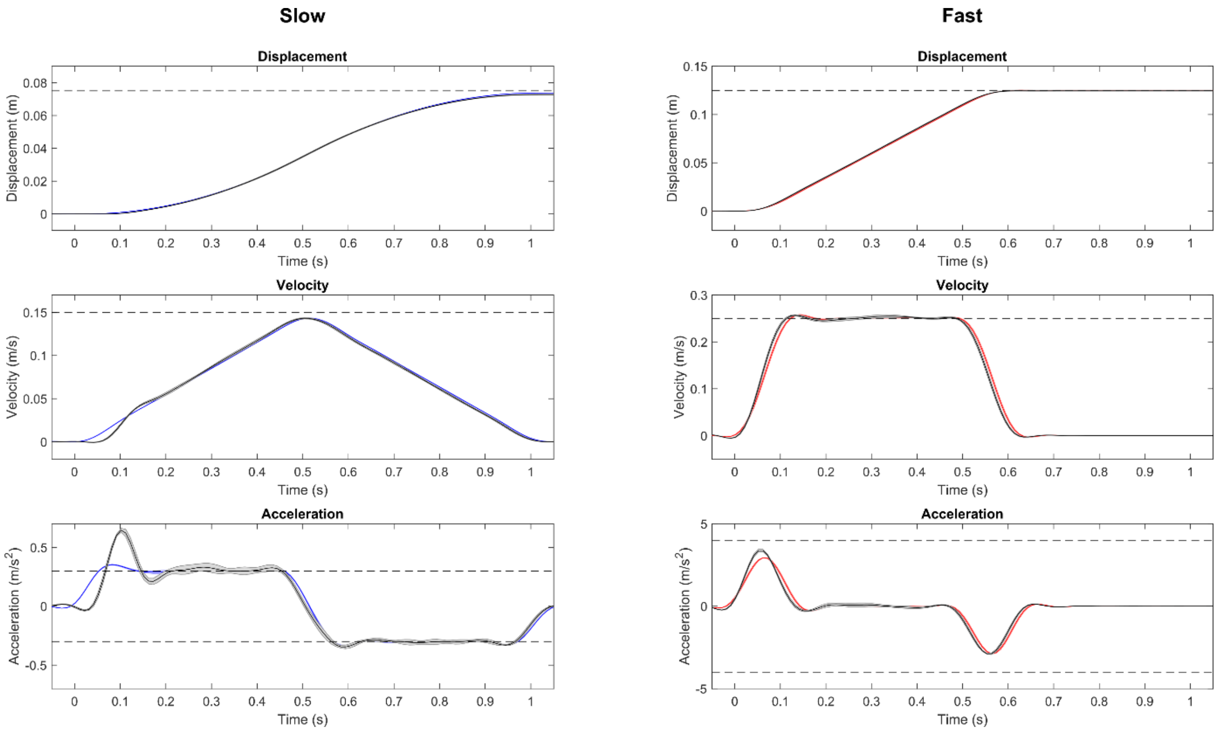

Supplementary Figure 1. Measured belt movement for the slow (left) and fast protocols (right) separately for the first (black) and subsequent nine perturbations in one measurement. The curves represent the mean and between-participant standard deviation (shaded area) of the measured belt movement. Dashed vertical lines show the target values of the peak displacement, velocity, and acceleration based on the programmed control signals of the belt. Time zero indicates the identified start of the perturbation. Note that the time scales are the same for both protocols, but vertical scales differ.

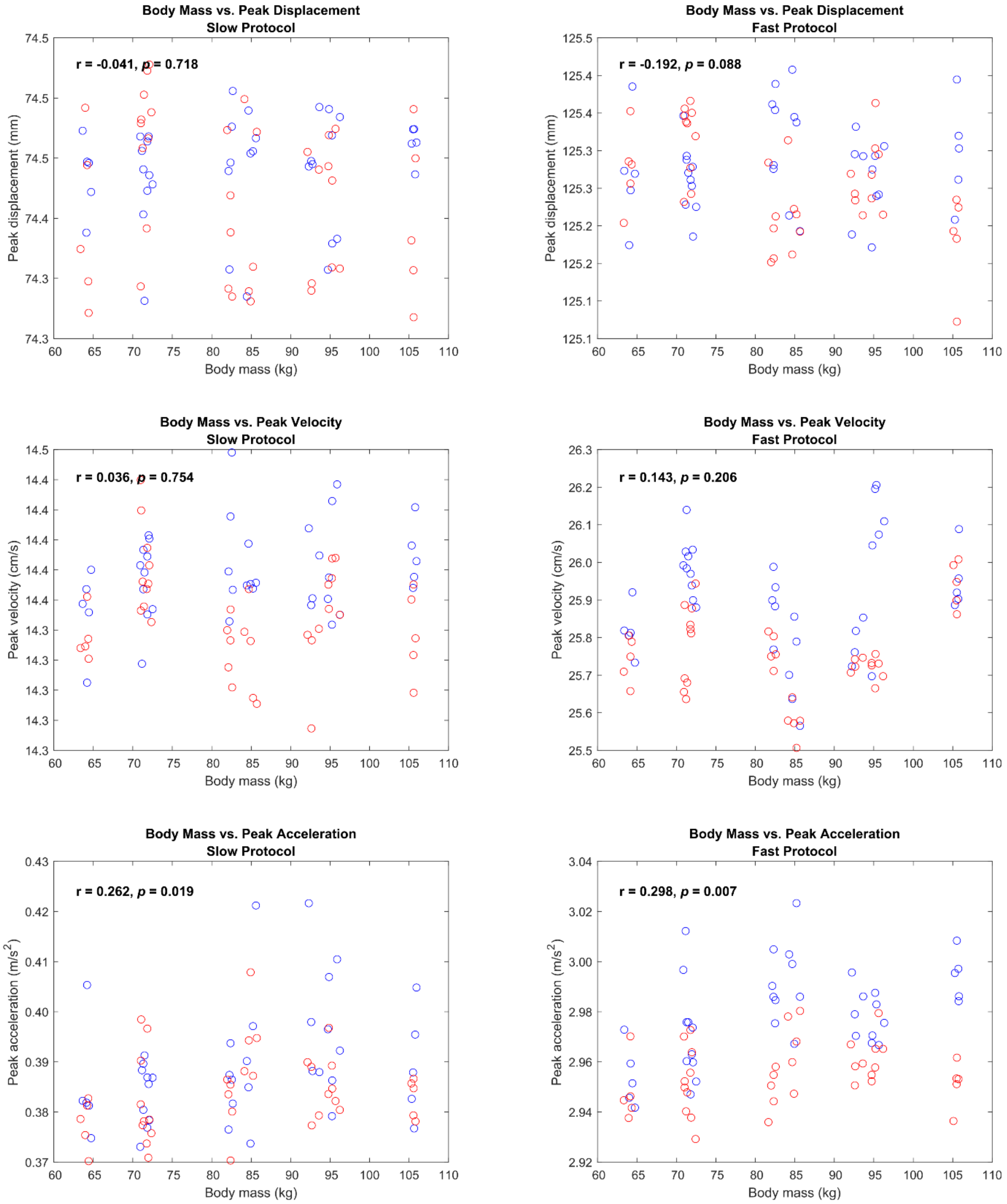

Supplementary Figure 2. Correlations between body mass and belt peak displacement, velocity, and acceleration. red symbols indicate data from backward perturbations and blue symbols from forward perturbations.

3 Habituation Effect

To investigate potential habituation effects (changes occurring acutely) we further analyzed the data from the day 1 slow backward perturbation (the first perturbations of the study). In this experimental setup, we utilized a practice set of perturbations before the actual measurement to reduce the effect of habituation on the measured data and we have considered also this practice set of perturbations in this additional analysis. We present below four figures: 1) a figure presenting COP trajectories for each individual perturbation and participant in the training set, 2) a figure presenting COP trajectories for each individual perturbation and participant in the actual measurement set, 3) average COP trajectories for training and measurement sets, and 4) average COP trajectory for each 9 perturbations in a set across the participants. Please note that we excluded the 2nd and 3rd perturbation from participant #7 due to a stepping response at the beginning of the training set.

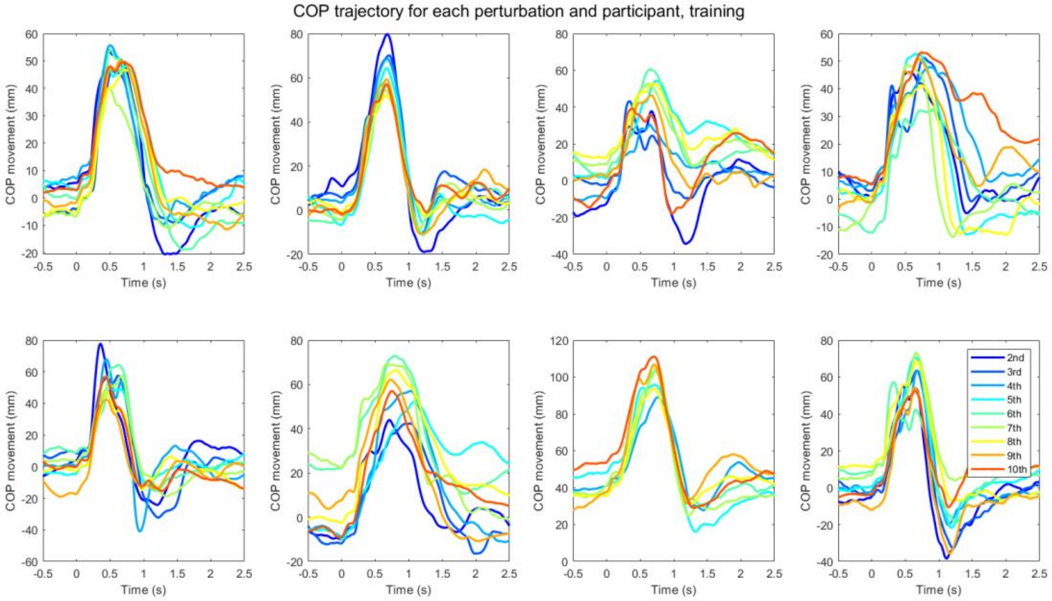

Supplementary Figure 3. COP trajectories for each perturbation and participant from the training set of day 1 in the slow backward perturbation protocol. Note the initial overcompensation in the balance recovery phase before the COP trajectory recovers to the baseline value.

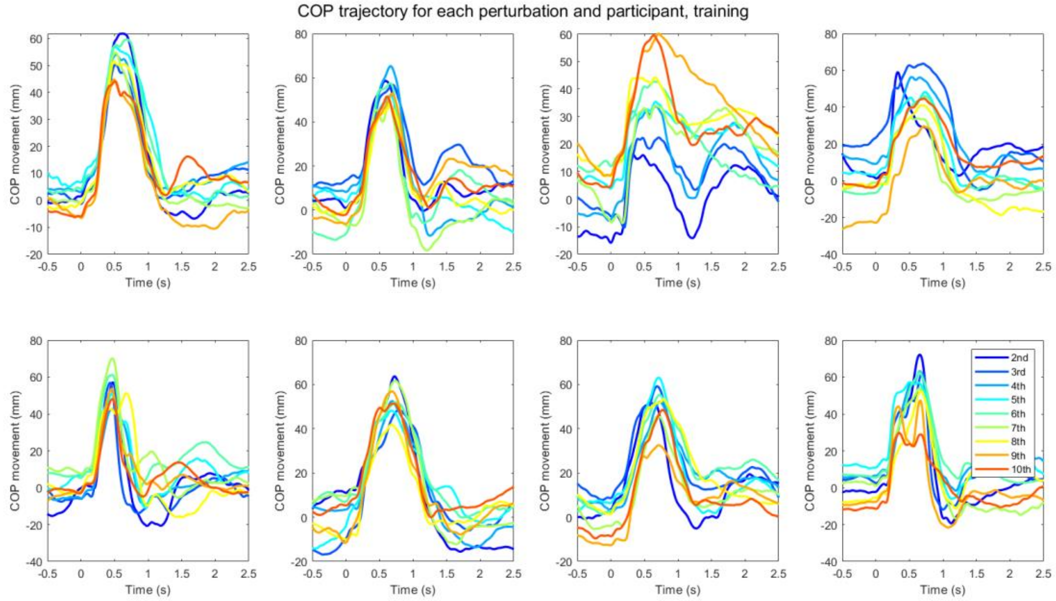

Supplementary Figure 4. COP trajectories for each perturbation and participant from the measurement set of day 1 in the slow backward perturbation protocol. Note that the overcompensation noted in the previous figure is not present.

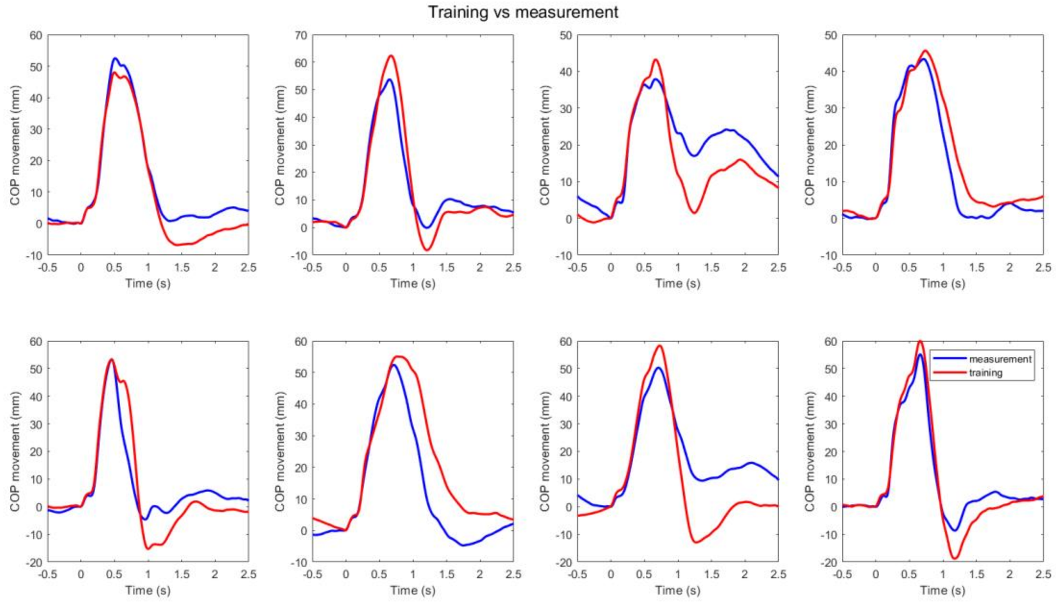

Supplementary Figure 5. Ensemble average COP trajectories from training and measurement sets of day 1 in the slow backward perturbation protocol highlighting the habituation effect within the participant.

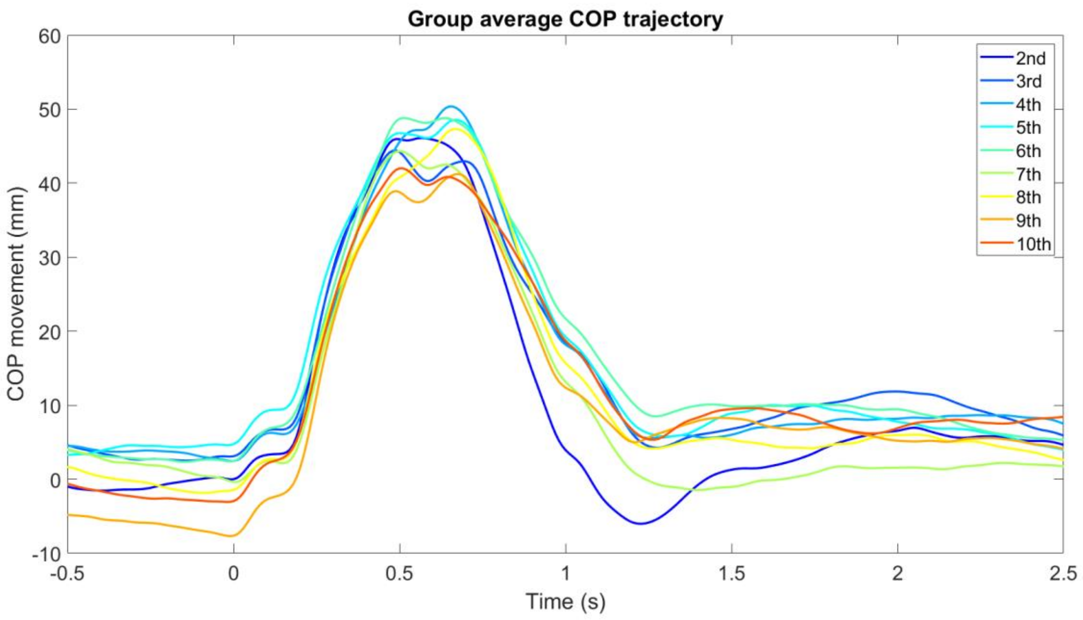

Supplementary Figure 6. Ensemble average COP trajectories from the measurement sets of day 1 in the slow backward perturbation protocol. Note that there are no apparent differences in the trajectories that would indicate habituation within the set. The only notable trend is the backward movement of the initial COP at the instant of perturbation onset.
